# Mapping the Prevalence of Lynch Syndrome in the Ceará—Northeast of Brazil

**DOI:** 10.1111/cge.70082

**Published:** 2025-09-27

**Authors:** Maria Claudia dos Santos Luciano, Paulo Goberlanio de Barros Silva, Rosane Oliveira de Sant'Ana, Clarissa Gondim Picanço de Albuquerque, Francisca Fernanda Barbosa Oliveira, Flavio da Silveira Bitencourt, Isabelle Joyce de Lima Silva Fernandes, José Fernando Bastos Moura, Maria Júlia Barbosa Bezerra

**Affiliations:** ^1^ Instituto Do Câncer Do Ceará/Faculdade Rodolfo Teófilo Fortaleza Brazil

**Keywords:** colorectal cancer, genetic evaluation, novel variant

## Abstract

Lynch syndrome (LS) is an autosomal dominant hereditary disorder that increases the risk of various cancers, especially colorectal (CRC) and endometrial cancer (EC). It results from pathogenic variants in mismatch repair (MMR) genes—primarily *MLH1*, *MSH2*, *MSH6*, and *PMS2*. Population‐specific variant frequencies emphasize the need for localized genetic studies. Methods This study investigated LS prevalence in Ceará, Northeast Brazil, analyzing 150 patients: 130 with CRC, 13 with endometrial cancer, and 7 with other tumors but a family history of LS‐associated cancers. Researchers used next‐generation sequencing (NGS) to examine 131 genes linked to hereditary cancer syndromes. Variants were classified as Lynch‐syndrome associated (MMR genes) or non‐Lynch‐associated (non‐MMR genes). Detection rates varied from 1.18 to 5.07 per 100,000 people; pathogenic variant prevalence ranged from 0 to 1.96 per 100,000 across microregions. Overall, the prevalence of MMR variants was 0.56 per 100,000, and 0.34 for non‐MMR variants. *MSH2* showed the highest number of pathogenic or likely pathogenic variants, followed by *MSH6*, *PMS2*, and *MLH1*. The study found a particular geographic distribution of LS‐related variants. One novel *MSH6* variant and two unreported non–Lynch variants (*APC* and *SMAD4*) were identified. Conclusions These findings highlight three novel variants in *MSH6*, *APC*, and *SMAD4*, and indicate that Ceará has a higher diversity and a unique spectrum of variants. This reinforces the importance of regional genetic screening and suggests the need to expand testing access, especially in high‐risk areas, to improve the early detection and prevention of hereditary cancers in Northeast Brazil.

## Graphical abstract

Distribution of pathogenic variants associated with Lynch syndrome across Brazil, with a particular emphasis on the state of Ceará. The illustration highlights the most frequently reported MMR genetic variants in Brazil and provides a magnified view of Ceará, where three novel variants were identified in the *APC*, *SMAD4*, and *MSH6* genes within specific microregions.

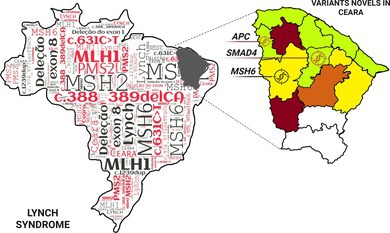

## 1 Introduction

Lynch syndrome (LS) is an autosomal‐dominant disorder caused by defective DNA mismatch repair (MMR) genes. It is associated with an increased risk of developing malignancies in multiple organs [1, 2]. Carriers of different pathogenic DNA mismatch repair (MMR) variants exhibit distinct patterns of cancer risk and survival as they age. Risk estimates for counseling, surveillance, and treatment planning should be tailored to each patient's age, gender, and specific pathogenic MMR variant [3]. LS is associated primarily with colorectal cancer (*CRC*) and endometrial cancer (*EC*). This elevated risk is linked to pathogenic variants in five genes (*MLH1*, *MSH2*, *MSH6*, *PMS2*, and *EPCAM*). Generally, *MLH1* or *MSH2* variants are associated with a higher risk of cancer compared to other genes [4]. A previous study correlated *MLH1* variants with the highest risk of colorectal cancer, and *MSH2* variants with the highest risk of other cancers [4].

The importance of this syndrome has been highlighted in numerous systematic reviews over the past three decades [5, 6, 7]. The evidence consistently indicates that registration and screening programs for CRC result in a reduction of its incidence and mortality in patients with LS [6]. Modifiable risk factors, such as diet and physical activity, have been identified or associated with the development of specific cancers, including EC and CRC, even in individuals with pathogenic variants [7].

Genetic databases allow comparisons based on intrapopulation specificities. The Genome Aggregation Database (gnomAD) [8] is a widely used resource of exome and genome sequencing data from unrelated individuals of diverse ancestries, collected from various disease‐specific and population genetic studies. In Brazil, the Genetic Repository of Variants (ABRAOM) was established to address the limited representation of the Brazilian population in global databases. ABRAOM [9] contains genomic variants from whole‐exome and whole‐genome sequencing, highlighting the importance of studying the unique Brazilian genetic background.

A meta‐analysis identified the genetic background of Brazilians as based on European, African, and Native American ancestry [10]. However, it is crucial to observe the differences, which indicate significant variations in ancestry. Brazil has a sizeable territorial extension with widespread miscegenation and unequal economic power across its regions. Ceará state, located in the Northeast of Brazil, has a population of 5,511,018 inhabitants distributed over 148,886 km^2^. This context highlights how admixture proportions can vary extensively among Brazilian populations and throughout Latin America. Additionally, there is a need for more studies in the Central‐West and North regions to capture a complete picture of Brazil's genomic ancestry. Historically, the ethnic distribution in Ceará is distinct from the national average and differs significantly from other states in the same region, such as Bahia. It is also important to emphasize the poor genetic representation of these specific populations in existing databases and published studies [8].

Therefore, genetic characterization in regions with poor genetic representation and limited economic power is essential. This work ensures that diverse populations are equitably represented in future genetic databases and research, rather than being underrepresented.

## 2 Results

The study population included 150 patients diagnosed with primary or secondary tumors of the colon (*n* = 130) or endometrium (*n* = 13). Additionally, seven patients with other tumor types were included based on a family history of colorectal and/or endometrial cancer. All patients were diagnosed in Ceará, a state in Northeast Brazil, between 2019 and 2021.

Patient characteristics are summarized in Table 1. Participants ranged in age from 20 to 78 years. Following NCCN guidelines for LS, age at diagnosis was categorized into two groups: < 50 years (57.3%) and ≥ 50 years (42.7%).

**TABLE 1:** Sociodemographic characteristics and comorbidities among patients with Lynch syndrome.

Age at diagnosis (years)	*n*	(%)
< 50	86	57.3
> 50	64	42.7
Sex		
Male	56	37.33
Female	94	62.67
Education level		
No schooling	5	3.33
Incomplete elementary school	43	28.67
Complete elementary school	18	12.00
Incomplete secondary (middle and high school)	12	8.00
Complete secondary (middle and high school)	43	28.67
Incomplete University	8	5.33
Complete University	17	11.33
Not informed	4	2.67
Diabetes	23	15.33
Hypertension	46	30.67
Body mass index (BMI)		
Bellow (< 18.8)	1	0.67
Normal weight (18.8–24.9)	59	39.33
Pre‐obesity (25–29.9)	60	40.00
Obesity (> 30)	29	19.33
Not informed	1	0.67
Other comorbidities	41	27.33

The majority were female (62.67%). In terms of education, over half (52%) had not completed secondary school or had a lower level of formal education. In contrast, among regional subgroups, patients with confirmed MMR variants were more likely to reside in the Fortaleza metropolitan area and have higher levels of education.

The most reported comorbidities were hypertension (30.67%), diabetes (15.33%), and obesity (19.33%) (Table 1). Additional comorbidities were observed in 27.33% of patients. A complete list of these conditions, which includes dyslipidemia, psychological disorders, and psoriasis, is provided in Table S1.

**FIGURE 1:**
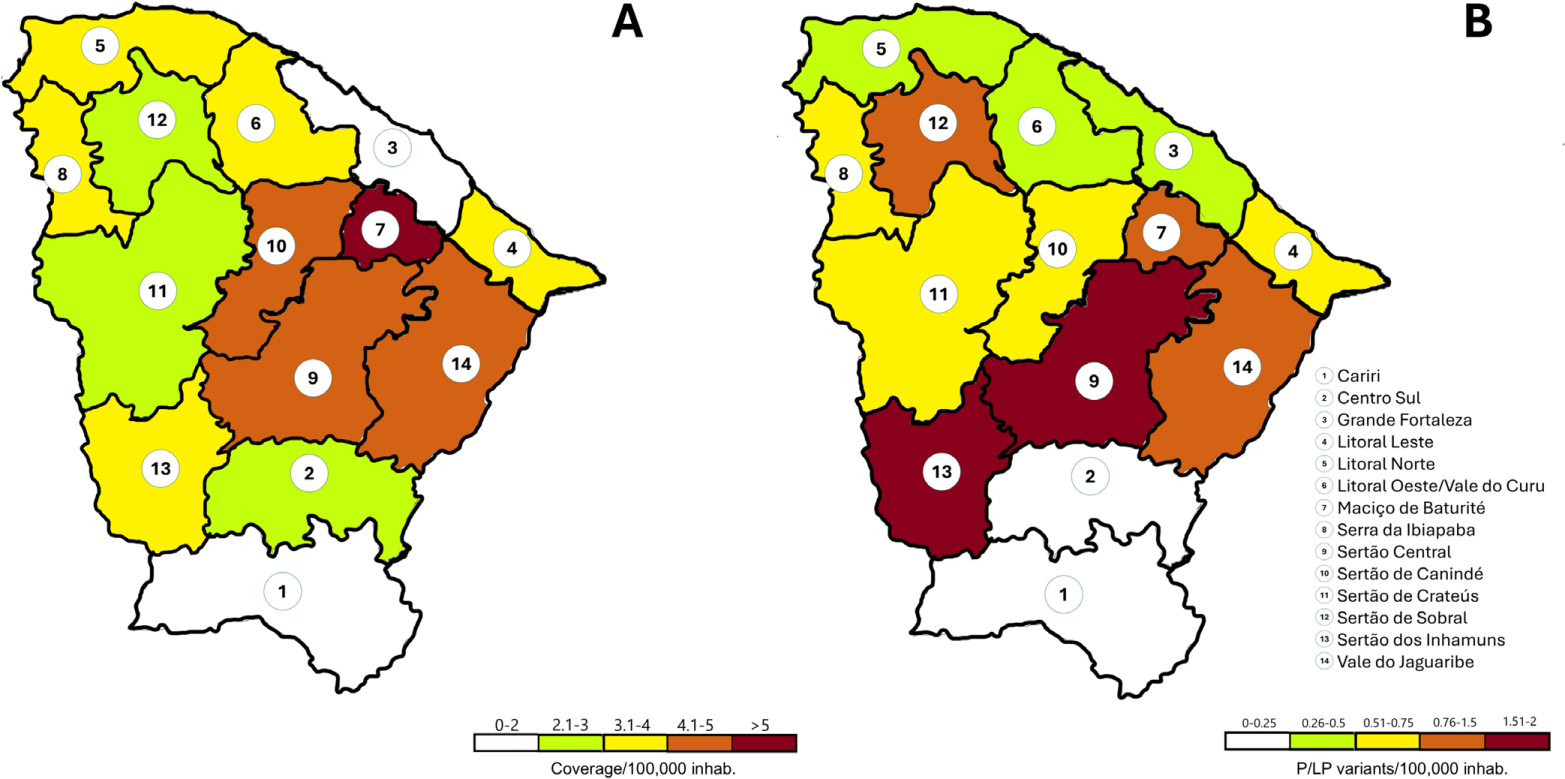
Spatial distribution maps of MMR pathogenic/likely pathogenic variants testing across microregions in Ceará, Northeast Brazil. (A) Average adjusted coverage of Lynch syndrome testing per 100,000 inhabitants. (B) Prevalence of Lynch syndrome pathogenic/likely pathogenic variants per 100,000 inhabitants. [Colour figure can be viewed at wileyonlinelibrary.com]

Patients were tested across 110 municipalities in the state of Ceará. The microregional distribution of the cohort, as depicted in Figure 1, was as follows: Sertão do Cariri (*n* = 14), Centro Sul (*n* = 11), Grande Fortaleza (*n* = 225), Costa Leste (*n* = 14), Costa Norte (*n* = 17), Costa Oeste (*n* = 26), Maciço de Baturité (*n* = 19), Serra de Ibiapaba (*n* = 14), Sertão Central (*n* = 33), Sertão de Canindé (*n* = 20), Sertão de Sobral (*n* = 21), Sertão de Crateús (*n* = 20), Sertão de Inhamuns (*n* = 9), and Vale do Jaguaribe (*n* = 33).

Test coverage for LS variant detection normalized was adjusted per 100,000 inhabitants, and the distribution across microregions was heterogeneous (Figure 1A). The detection rates ranged from 1.18 to 5.07, with lower rates observed along the coast and higher rates in the central regions.

The prevalence of pathogenic variants across the state varied from 0 to 1.96 per 100,000 inhabitants. This pattern mirrored the heterogeneous distribution of test coverage in the tested municipalities, with microregion 13 (Sertão de Inhamuns) reporting a prevalence of 1.63 variants per 100,000 (Figure 1B). The geographic distribution of the ratio of founded variants to tested individuals is shown in Figure S1.

The statewide prevalence per 100,000 inhabitants was 0.56 for MMR genes and 0.34 for non–MMR genes (Table S2). As expected, statistical analysis revealed a significant correlation between testing coverage and the frequency of detected pathogenic variants (Figure S2). Tables S2 and S3 provide a detailed description of coverage and prevalence by microregion and municipality.

**FIGURE 2:**
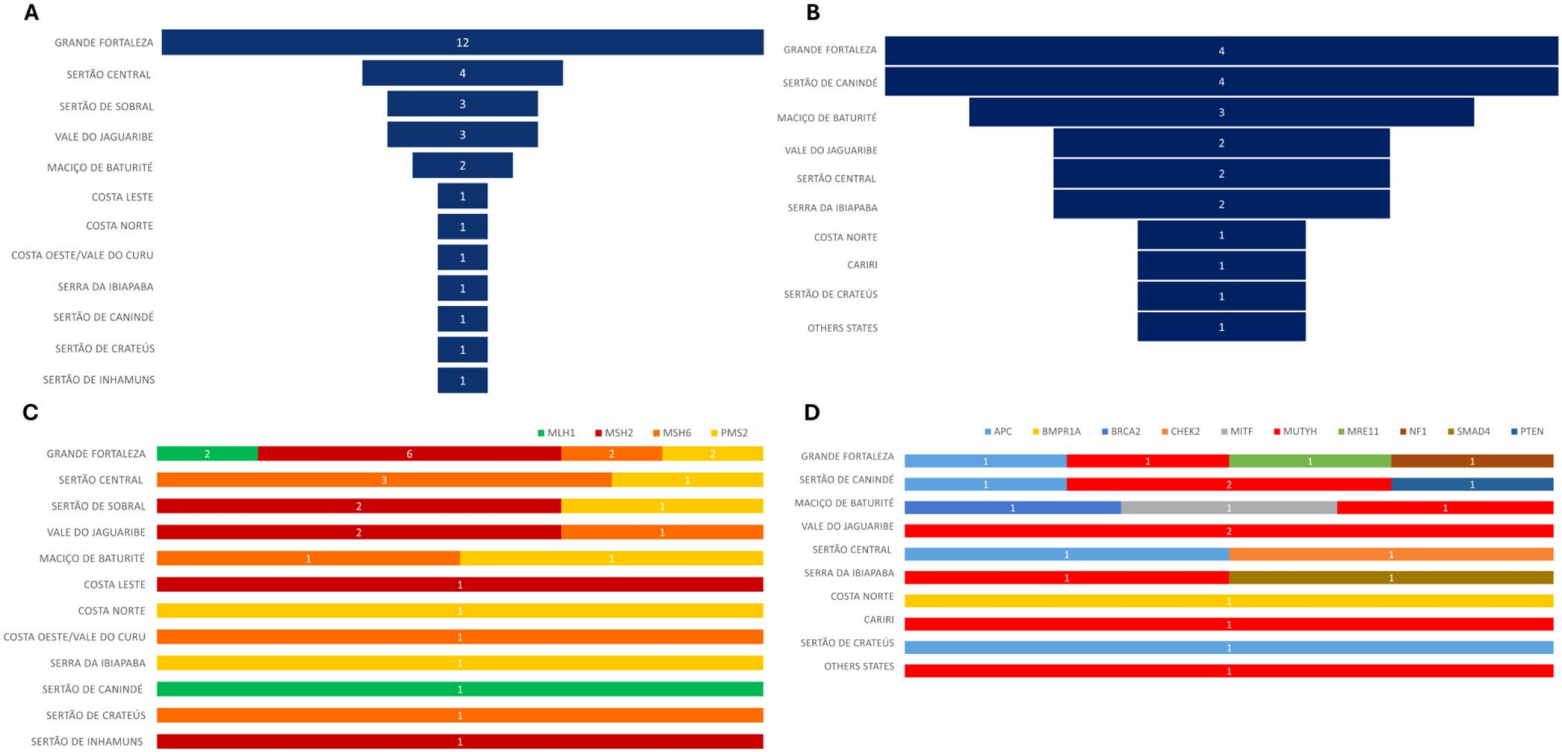
Geographic distribution of pathogenic/likely pathogenic variants across microregions in Ceará. (A) Total number of variants in MMR genes per microregion. (B) Distribution of variants by specific MMR gene per microregion. (C) Total number of variants in non‐MMR genes per microregion. (D) Distribution of variants by specific non‐MMR gene per microregion. [Colour figure can be viewed at wileyonlinelibrary.com]

A total of 50 pathogenic or likely pathogenic variants were identified. Of these, 31 variants were associated with LS, in MMR gene (Figure 2A) and 19 were not (Figure 2B). Among the MMR genes, the most frequently affected were *MSH2* (*n* = 11), *MSH6* (*n* = 10), *PMS2* (*n* = 7), and *MLH1* (*n* = 3) (Figure 2C). For non‐MMR genes, variants were most commonly found in *MUTYH* (*n* = 10) and *APC* (*n* = 4) (Figure 2D).

The highest number of pathogenic/likely pathogenic variants, spanning both gene categories, was found in patients from the Fortaleza metropolitan area. Most individuals carrying these variants had colorectal cancer (*n* = 26). Notably, many patients with pathogenic variants also presented with multiple primary tumors (*n* = 18) (Table S4). As expected, the most common types of variants identified were frameshift or nonsense variants resulting from deletions, insertions, or indels. These were frequently followed by missense variants, copy number variations (CNVs), and splice‐site alterations.

**FIGURE 3:**
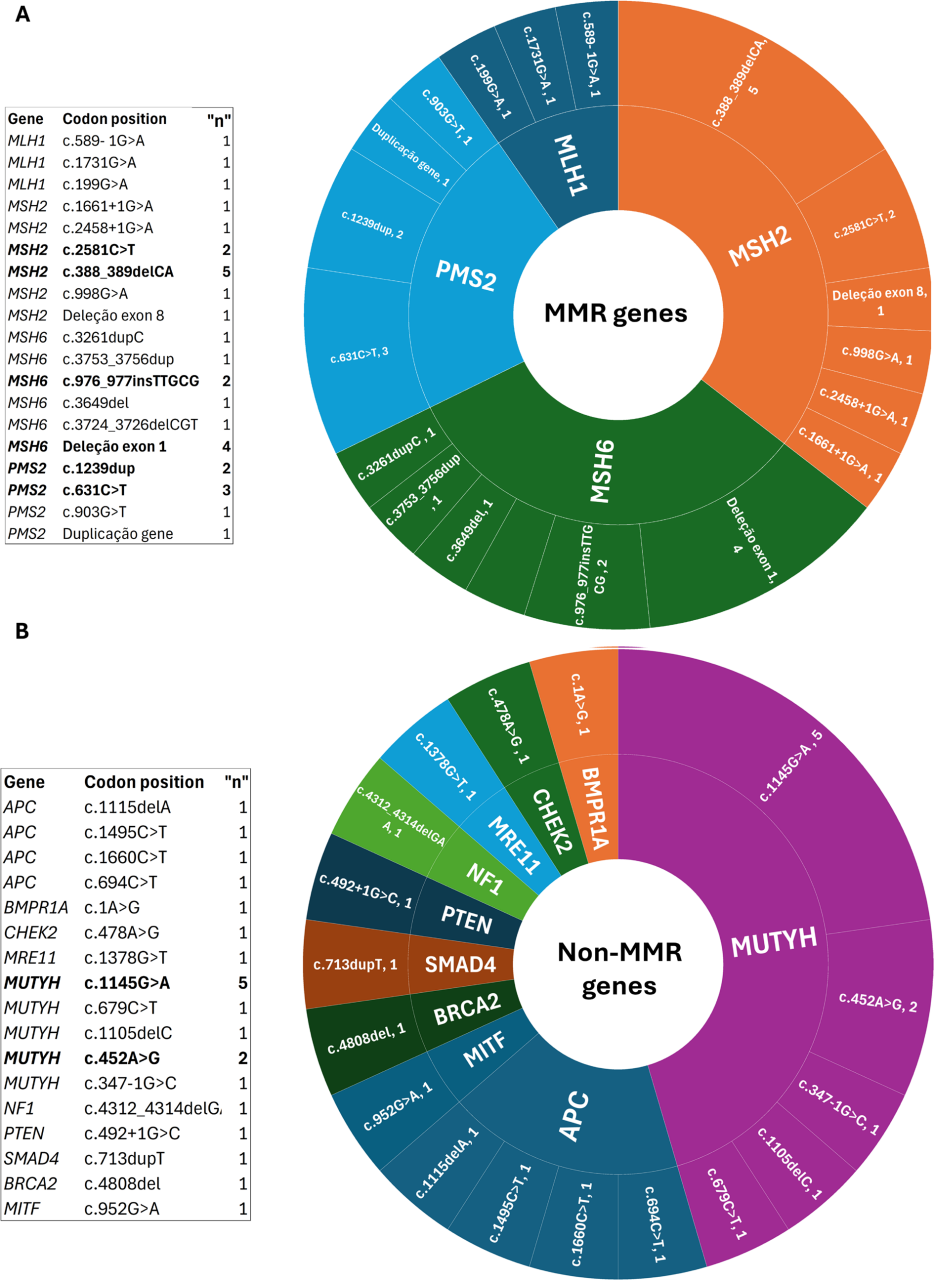
Distribution of pathogenic/likely pathogenic variants by gene category. (A) Frequency and classification of variants in MMR genes. (B) Frequency and classification of variants in non‐MMR genes. [Colour figure can be viewed at wileyonlinelibrary.com]

The most frequently observed MMR variant was *MSH2* (Clinvar ID: 91090, *n* = 5), followed by an exon 1 deletion in *MSH6* (*n* = 4) and *PMS2* (Clinvar ID: 234508, *n* = 3) (Figure 3A). Additionally, the novel variant in *MSH6*, NM_000179.3:c.3649del (p.Arg1217Glufs*11), was identified in one patient with colorectal cancer (Table S5).

Among non–MMR genes, the most frequent variants were two missense variants in *MUTYH* (Clinvar ID: 5294, *n* = 5) and (Clinvar ID: 5293, *n* = 3) (Figure 3B). Two novel variants were also identified in non‐MMR genes: *SMAD4* NM_005359.6: c.713dup (p.Leu238Phefs*26) and *APC* NM_000038.6: c.1115delA (p.Asn372Ilefs*82). The *MUTYH* (Clinvar ID: 5294) variant was identified in both homozygous and compound heterozygous states. The latter was determined in combination with the pathogenic *MUTYH* (Clinvar ID: 5293) and *MLH1* (Clinvar ID: 941211) variants (Table S6). The distributions of all variants by gene and microregion are detailed in Tables S5 and S6. The zygosity of *MUTYH* variants and the occurrence of multiple variants across the microregions are described in Table S7.

## 3 Discussion

The prevalence of pathogenic/likely pathogenic (P/LP) variants in MMR genes was 0.56 per 100,000 inhabitants in Ceará. P/LP variants constituted 33.33% (*n* = 50) of our population, with the breakdown being 20.66% in MMR genes and 12.66% in non‐MMR genes. Among the MMR genes, the frequency was distributed as follows: *MLH1* (9.68%), *MSH2* (35.48%), *MSH6* (32.26%), and *PMS2* (22.58%).

The Global Cancer Observatory [11] indicates the following incidence rates per 100,000 inhabitants: 10.7 for colon, 7.1 for rectum, 0.54 for anus, and 8.4 for corpus uteri, excluding P/LP variants. LS is one of the most prevalent hereditary cancer syndromes in humans and accounts for approximately 3% of unselected patients with CRC or EC [2], which has a significant effect on global incidence and mortality.

The analysis of SNV variant frequency in health public databases indicates a higher number of variants identified in the ABRAOM database (10,717) than in gnomAD v2.1 (6,294), across all MMR genes and regardless of their classification (Table S8). It is essential to mention the differences observed in the number of variants in the databases for specific MMR genes, especially for *MSH6* (882 vs. 1,897) and *MSH2* (7,245 vs. 1,707) in the ABRAOM and gnomAD v2.1 databases, respectively. These genetic differences from the other populations highlight the relevance of this syndrome in Brazil.

The number of studies evaluating the overall prevalence of LS remains low. However, it is important to highlight the difference in the prevalence of MMR variants between EC and CRC. A review of various worldwide studies shows that the prevalence of pathogenic/likely pathogenic (P/LP) variants is comparable to other populations. A meta‐analysis across various ethnic, geographic, and clinical populations of multiple studies demonstrated a prevalence of LS across various ethnic, geographic, and clinical populations. However, the authors noted significant heterogeneity in the overall prevalence. The analysis indicated a prevalence range of 1.1% to 5.1% for the MSI and CRC groups, respectively [12]. A study evaluating CRC patients in the US found a total prevalence of 16% for patients with pathogenic/likely pathogenic variants and 10.6% for MMR‐deficient patients [13].

Specifically for EC, Lu et al. (2006) [14] found a 9% prevalence of LS without regard to MMR status. In contrast, Post et al. (2021) [15] reported a prevalence ranging from 2.8% to 9.5% when evaluating groups with and without MMR proficiency. These observed variations in CRC and EC suggest that different factors, including the analyzed population and the tumor characteristics, could influence prevalence.

The prevalence of pathogenic variants across the state ranged from 0 to 1.96 variants per 100,000 people. Notably, Microregion 13 showed a high prevalence of 1.63 variants per 100,000 people, despite having a median coverage for the area. The distribution of these variants across MMR genes and non‐MMR genes highlights the necessity of efforts to characterize this population. The identification of three novel genetic variants (*MSH6*, *APC*, and *SMAD4*), even with low coverage in some regions (Sertão de Cratéus), underscores the importance of ethnic diversity and the poor genetic representation in databases and published studies [8].

In the context of regionalizing variant mapping across the state, it is relevant to highlight the copy number variants (CNVs) found, such as the *PMS2* duplication (Costa Norte, *n* = 1), the *MSH6* exon 1 deletion (Costa Oeste/Vale do Curu, *n* = 1; Sertão Central, *n* = 1; Vale do Jaguaribe, *n* = 2), and the *MSH2* exon 8 deletion (Sertão de Sobral, *n* = 1). These CNVs were found predominantly in regions of the state with low testing coverage and outside the metropolitan area, which indicates the need for a higher number of subjects to be investigated in this population to determine the actual relevance of these variants. The presented positive correlation graphics support the necessity of increased testing to characterize this population properly.

The copy number variants (CNVs) (> 50 bps) in LS are broadly analyzed in oncological contexts (Table S9). Our study identified three different CNVs, with the most frequent in our cohort being an *MSH6* exon 1 deletion, highlighted in the Vale do Jaguaribe microregion, with two cases. The challenges and importance of CNVs in LS have been widely reported in the literature [16, 17], which underscores the importance of evaluating deep‐intronic sequences [18] and CNV‐neutral structural genomic rearrangements [19].

In the context of single‐nucleotide variants (SNVs), a higher absolute number of variants in the Fortaleza area was expected, considering the high number of inhabitants in this metropolitan region. Even with a high absolute number, this region did not present high coverage or prevalence of pathogenic variants; however, it is responsible for 38.7% of the found MMR variants (Table S4) and 26.3% of non‐MMR variants (Table S5). The most frequent variant was the frameshift variant *MSH2* (Clinvar ID 91090, *n* = 5); it is a variant with higher prevalence in affected individuals, reported mainly in different ethnic groups such as German [20], Brazilian [21], Chile [22], Australian [23], Argentinean [24], and Portuguese [25], compared to its absence in the control database (gnomAD, v2.1). The second most frequent SNV was *PMS2*: c.631C>T (*n* = 3), which has been identified in LS patients in French [26], Australian [27], and China [28, 29].

The other three variants identified in more than one patient were the *MSH2*: c.2581C>T (*n* = 2), present in German [20]. and French [30] studies with CRC cancer. The *PMS2*: c.1239dup seems more frequent in the Brazilian population; it was present in Latin America [31] and the Brazilian [32] populations. The *MSH6*: c.976_977insTTGCG, on the other hand, was not found in previous publications; however, it has earlier entries in clinical databases in patients with endometrial carcinoma and LS (Clinvar ID: 1685956). The variant in *MSH6*, NM_000179.3:c.3649del (p.Arg1217Glufs*11), found in a patient from Sertão de Crateús, was not previously reported in any database.

For the non‐MMR genes, we identified two novel variants that were not previously reported in clinical databases: one in *APC* (c.1115delA) and one in *SMAD4* (c.713dupT). These variants were found in patients from the Grande Fortaleza area, Sertão de Canindé, and Serra da Ibiapaba. It is essential to mention that these microregions had high coverage and intermediate coverage, respectively.

The historical conditions of this population highlight two critical issues: the difficulty of accessing genetic testing and the elevated frequency of pathogenic/likely pathogenic (P/LP) variants. The higher prevalence of P/LP variants (33% overall and 20.6% in MMR genes), along with the discovery of three novel variants even in regions with poor coverage, reinforces the importance of studying underrepresented populations to determine the full mismatch repair gene variant spectrum. The positive correlation between testing and detection across different microregions supports the need for health policies.

## 4 Study Limitations

A limitation of our study was the inability to evaluate patients from all municipalities, which necessitated grouping them into the state's administrative macro‐regions. The public health public Brazilian system divides some patients into different health coverage areas, and some patients are referred for treatment to hospitals closer to their homes, especially those in the southern part of the state. This is the first state‐level survey of LS, which even encouraged our center's participation in the national screening of cancer predisposition syndromes. This initiative could serve as guidance for small and medium‐sized cancer centers on how to integrate into broad genetic profiling networks in their countries.

The limited number of worldwide studies on the prevalence of hereditary cancer predisposition syndromes highlights the need for each study to report not only its mutation frequency, but also its relationship with the studied population and the associated cancer types.

## 5 Conclusions

In summary, this study demonstrates a geographic and clustered distribution of LS variant prevalence. It further highlights the spatial risk of these variants in certain microregions of the state. Our study identified one novel variant in the MMR gene *MSH6* and two others in non‐MMR genes, one in *APC* and one in *SMAD4*. Preventative strategies, such as increasing genetic testing in this region, can be targeted to high‐risk counties based on the observed prevalence. Such findings will help inform the establishment of novel surveillance systems to raise awareness of potential cancer prevention measures in populations living in these high‐risk microregions.

## 6 Materials and Methods

### 6.1 Area and Study Sample

The study area included 14 counties in the state. County boundary data downloaded from the Brazilian Census were used for mapping and helped determine the function estimation (Figure 1).

This study included all patients evaluated for LS according to the NCCN criteria between June 2018 and June 2022 at the ICC. All the considered NCCN criteria are listed in Table S11. Patients who did not meet any inclusion criteria were excluded from the study. LS was investigated as a prospective cohort study involving 150 consecutive patients diagnosed with either colorectal or endometrial cancer at Haroldo Juaçaba Hospital, Ceará, Brazil. The primary objective was to determine the prevalence of LS.

### 6.2 Data Collection

Baseline data on demographics and personal and family history of cancer, including histopathology reports, were collected at enrollment or during the initial consultation with the genetic counselor. Additional information, such as socio‐demographic characteristics, including microregion of birth, educational level, monthly income, tobacco use, and alcohol consumption, was obtained from all participants. All patients provided written informed consent after receiving an explanation of the study, and blood samples were collected. Plasma was separated and stored at −20°C until analysis.

Patients who had not attended the genetics clinic within the previous 12 months were contacted, and any updates to their cancer history were recorded. Follow‐up time was defined as the period from detecting the first index cancer and entering the LS investigation to the last follow‐up date or date of death.

### 6.3 Genetic Variant Testing and Immunohistochemistry

The methods used for germline variant analysis have been previously described by Kraus et al. [33]. Genomic DNA was extracted from peripheral blood and subjected to direct sequencing. A panel of 131 genes associated with hereditary cancer syndromes was analyzed (Table S10).

The variants were categorized as Benign (B), Likely Benign (LB), Variant of Uncertain Significance (VUS), Likely Pathogenic, or Pathogenic, following ACMG 2015 guidelines [34], considering different criteria such as population frequency, functional studies, segregation, de novo occurrences, allelic data, phenotype data, and *in silico* predictions. Genetic variants were classified as MMR genes (variants in *MSH6*, *MLH1*, *MSH2*, and *PMS2*) or non‐MMR genes (other pathogenic or likely pathogenic variants). Gene classification was based on definitive associations with LS as defined by ClinGen (MONDO:0005835). All variants were annotated using the hg19 human genome reference build. VUS, LB, and B were not considered to be distributed over microregions.

When tumor tissue was available, immunohistochemistry (IHC) staining was performed to determine the expression of *MLH1*, *MSH2*, *MSH6*, and *PMS2* proteins after the histopathologic diagnosis.

### 6.4 Analysis and Statistics

The patient population was characterized using descriptive statistics, including mean, standard deviation, median, and range for continuous variables and frequencies for categorical variables.

For the calculation of overall test coverage for LS variant detection (tests per 100,000 inhabitants) and the overall prevalence of pathogenic variants (P/LP variants per 100,000 inhabitants), we used official population data from the Brazilian Institute of Geography and Statistics (IBGE) [35]. Data normalization was performed by aggregating the data for each patient's municipality of origin across the microregions for the year of testing. Additionally, the prevalence of P/LP variants among colorectal cancer (CRC) [36] patients was calculated based on the total number of estimated CRC cases in Ceará, as reported by the National Cancer Institute of Brazil (INCA).

A separate calculation was also performed to determine the total prevalence for each microregion, considering all individuals in that microregion regardless of their municipality of origin. We used official population data from the Brazilian Institute of Geography and Statistics (IBGE) [37].

To standardize the P/LP variant rate, the number of positive patients (with a LS pathogenic/likely pathogenic variant) was divided by the tested population of each municipality, as officially estimated by the IBGE. Fisher's exact test was employed to compare the prevalence of MMR gene variants among patients diagnosed with LS.

## Conflicts of Interest

The authors declare no conflicts of interest.

## Supporting information


**Figure S1:** Spatial distribution map of the proportion of individuals with Pathogenic/Likely Pathogenic (P/LP) MMR variants among those tested (%), across the microregions of Ceará, Northeast Brazil.

**Figure S2:** Statistical correlation between the number of individuals with Pathogenic/Likely Pathogenic (P/LP) variants and the number of individuals tested, with values adjusted per 100 000 inhabitants.

**Table S1:** A list of comorbidities and associated conditions among study participants with Lynch syndrome
**Table S2:** Assessment of the Coverage, Prevalence of Pathogenic/Likely Pathogenic Genetic Variants, and ratio of P‐LP variant over tested in Microregions of Ceará, Northeast Brazil
**Table S3:** Assessment of the number of patients with Pathogenic/Likely Pathogenic Genetic Variants in MMR genes, per city
**Table S4:** Summary of the presence of Pathogenic/Likely Pathogenic (P/LP) variants in MMR and non‐MMR genes, their association with loss of immunohistochemical (IHC) staining for mismatch repair proteins, and the associated cancer types and tumor count
**Table S5:** Assessment of Pathogenic/Likely Pathogenic (P/LP) variants in MMR genes per microregion in Ceará‐Northeast of Brazil, description of variant type, and HGVS nomenclature
**Table S6:** Assessment of Pathogenic/Likely Pathogenic (P/LP) variants in non‐MMR genes per microregion in Ceará‐Northeast of Brazil, description of variant type, and HGVS nomenclature
**Table S7:** Assessment of patients with two Pathogenic/Likely Pathogenic (P/LP) variants and their zygosity, categorized by gene and microregion in Ceará, Northeast Brazil
**Table S8:** Analysis of SNV (Single Nucleotide Variation) frequency in healthy (gnomAD v2.1 and ABRAOM) and affected (ClinVar) populations, using public databases
**Table S9:** Analysis of CNV (Copy Number Variants) frequency in healthy (gnomAD SV v2.1) and affected (ClinVar) populations, using public databases
**Table S10:** Genes are tested in the genetic panel for the identification of hereditary syndromes
**Table S11:** Inclusion criteria based on the NCCN 2018 guidelines for patients at risk for Lynch Syndrome (LS).

## Data Availability

The data that support the findings of this study are available from the corresponding author upon reasonable request.
