# Supplementary figures and images for: Mapping the Prevalence of Lynch Syndrome in the Ceará—Northeast of Brazil
Source: Clin Genet. 2025 Sep 27;109(4):630–8. doi: 10.1111/cge.70082 (PMC12958010; doi:10.1111/cge.70082)

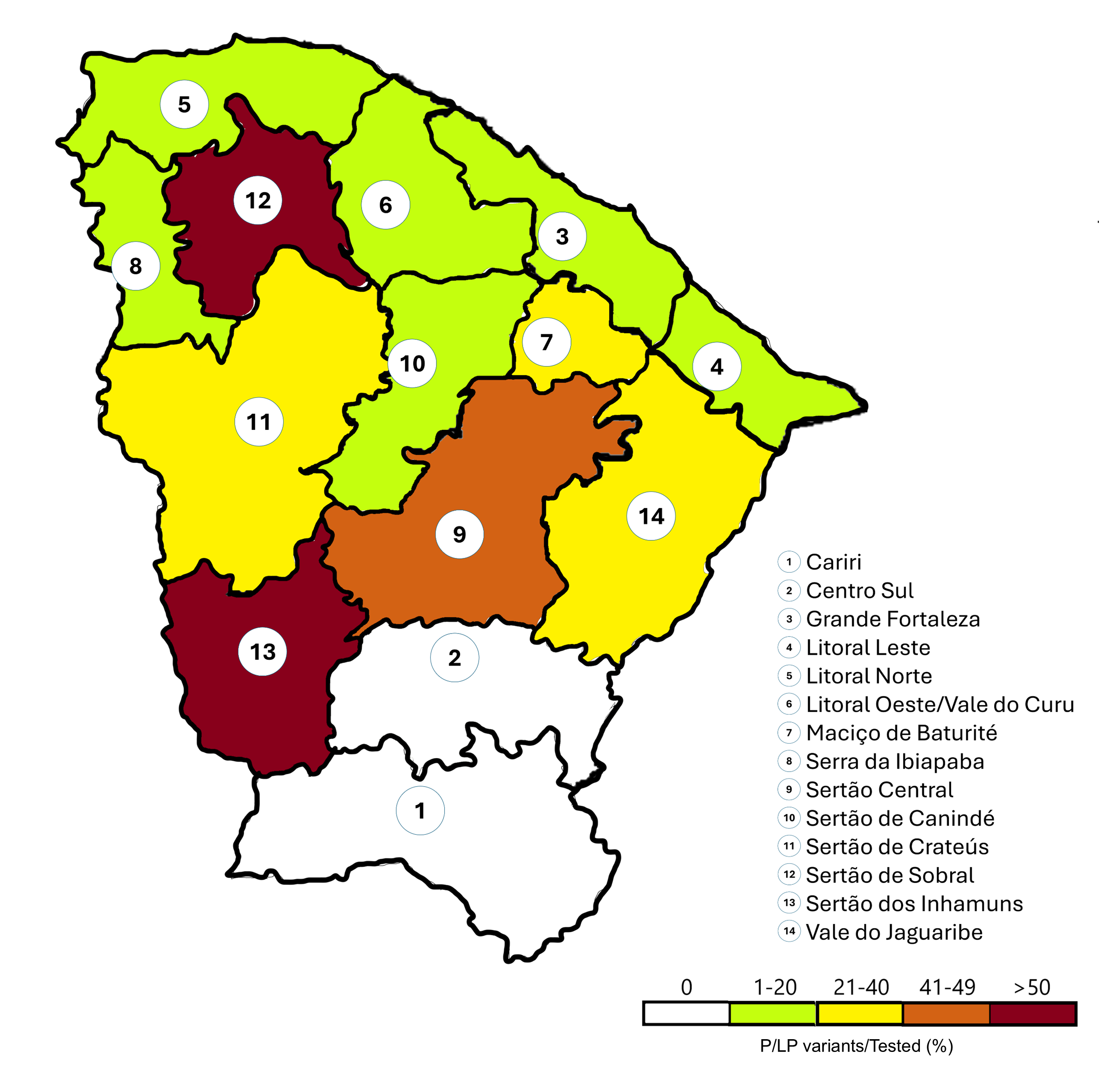

Supplement: Supplementary file 1 — Figure S1: Spatial distribution map of the proportion of individuals with Pathogenic/Likely Pathogenic (P/LP) MMR variants among those tested (%), across the microregions of Ceará, Northeast Brazil. [file CGE-109-630-s001.png]
