# Supplementary material for: Mapping the Prevalence of Lynch Syndrome in the Ceará—Northeast of Brazil
Source: Clin Genet. 2025 Sep 27;109(4):630–8. doi: 10.1111/cge.70082 (PMC12958010; doi:10.1111/cge.70082)
Supplement: Supplementary file 2 — Figure S2: Statistical correlation between the number of individuals with Pathogenic/Likely Pathogenic (P/LP) variants and the number of individuals tested, with values adjusted per 100 000 inhabitants. [file CGE-109-630-s002.pdf]

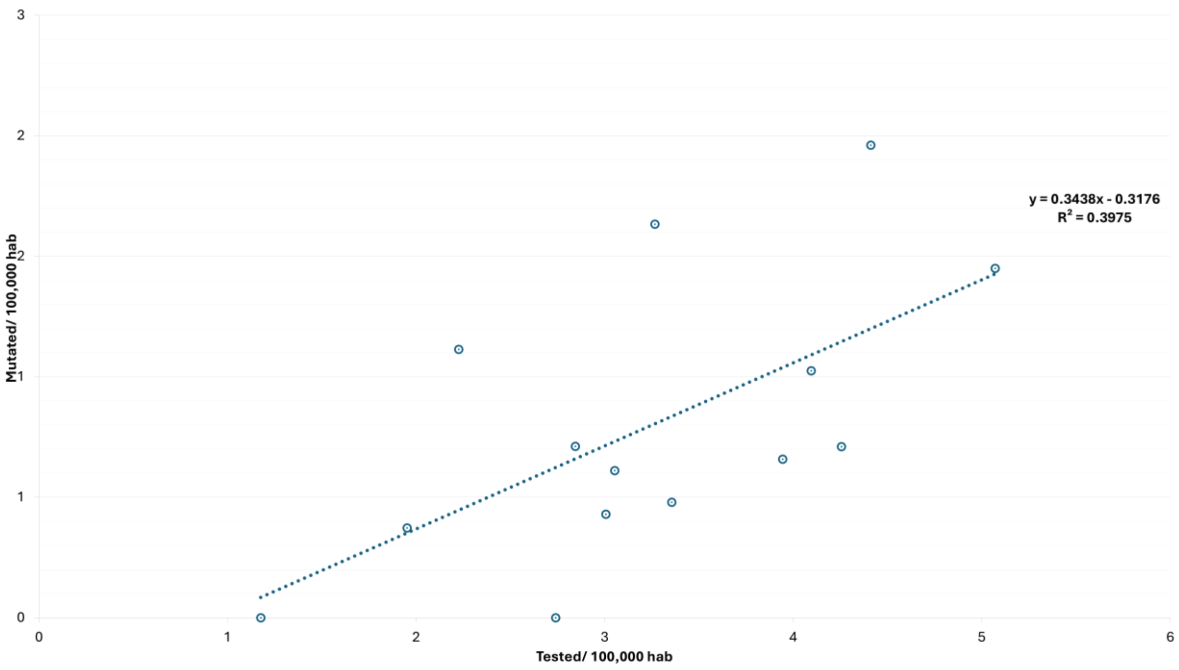

Supplementary Figure 2. Statistical correlation between the number of individuals with pathogenic/likely pathogenic variants and the number tested, adjusted per 100,000 inhabitants.
